# The value of marsh restoration for flood risk reduction in an urban estuary

**DOI:** 10.1038/s41598-024-57474-4

**Published:** 2024-03-21

**Authors:** Rae Taylor-Burns, Christopher Lowrie, Babak Tehranirad, Jeremy Lowe, Li Erikson, Patrick L. Barnard, Borja G. Reguero, Michael W. Beck

**Affiliations:** 1 University of California Santa Cruz, Santa Cruz, CA USA; 2 U.S. Geological Survey, Pacific Coastal and Marine Science Center, Santa Cruz, CA USA; 3 San Francisco Estuary Institute, Richmond, CA USA

**Keywords:** Climate change, Civil engineering, Climate-change adaptation

## Abstract

The use of nature-based solutions (NBS) for coastal climate adaptation has broad and growing interest, but NBS are rarely assessed with the same rigor as traditional engineering solutions or with respect to future climate change scenarios. These gaps pose challenges for the use of NBS for climate adaptation. Here, we value the flood protection benefits of stakeholder-identified marsh restoration under current and future climate change within San Francisco Bay, a densely urbanized estuary, and specifically on the shores of San Mateo County, the county most vulnerable to future flooding in California. Marsh restoration provides a present value of $21 million which increases to over $100 million with 0.5 m of sea level rise (SLR), and to about $500 million with 1 m of SLR. There are hotspots within the county where marsh restoration delivers very high benefits for adaptation, which reach $9 million/hectare with likely future sea level and storm conditions. Today’s investments in nature and community resilience can result in increasing payoffs as climate change progresses and risk increases.

## Introduction

Climate change is raising sea levels and increasing the threat of coastal flooding. By 2100, sea level is expected to rise 0.6–2.2 m along the contiguous United States, primarily due to the melting of ice sheets and glaciers, and thermal expansion of seawater^1^, which will significantly increase coastal flooding^2,3^. Flooding due to sea-level rise (SLR) is amplified by storms, which drive higher coastal water levels via surge, waves, and increased river discharge^4^. Increasing coastal population density further compounds the consequences of flooding. More than 600 million people live in the coastal zone, a number which is expected to increase to more than 1 billion by 2050^5^. These factors combine to create high and rising flood risk on coasts around the world.

Recent research suggests that in California, 675,000 people and $250 billion in property are at risk of flooding in a scenario with 2 m of sea level rise combined with a 100-year storm^4,6^. The population bordering San Francisco Bay (“the Bay Area”) accounts for two-thirds of future flooding impacts in California, and the cost of raising Bay area coastal protection structures to prepare for 2 m of SLR could reach $450 billion^7^. Across California and the West Coast of the United States West Coast, the communities bordering low-lying urban estuarine environments including Puget Sound, San Francisco Bay, and smaller Southern California lagoons and estuaries are all highly vulnerable to flooding, resulting in the majority of socioeconomic exposure of the U.S. West Coast^4^.

**Figure 1:**
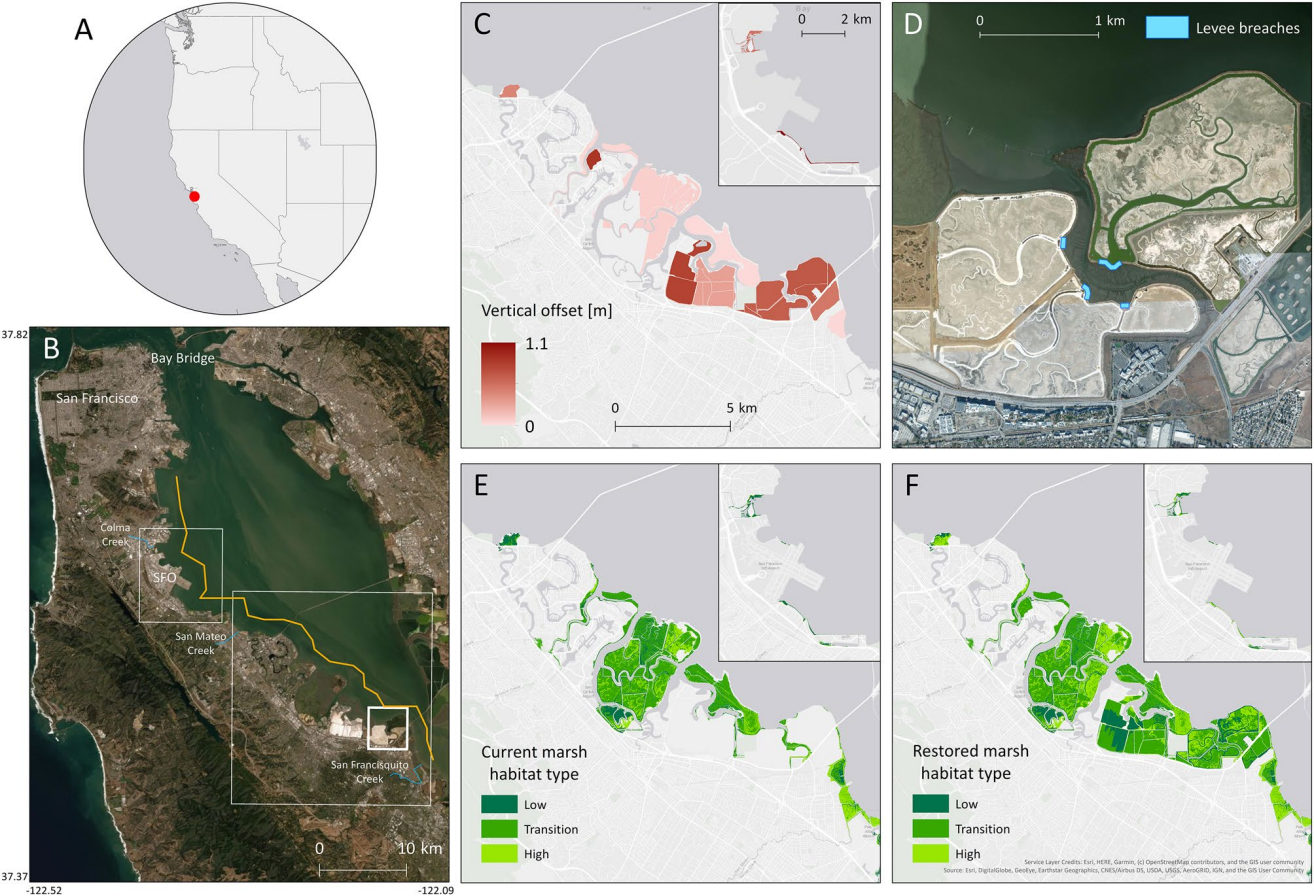
Location of the study area and details of the simulated restorations. (**A**) and (**B**) Location of this study, shown by the red dot in (**A**) and the orange line in (**B**). The thin white boxes in (**B**) show the extent of the maps in (**C**), (**E**) and (**F**) and the thick white box in (**B**) shows the extent of the map in (**D**). (**C**) Vertical offset applied to the restoration sites to simulate sediment accretion/nourishment, bringing each site to + 1 m relative to mean sea level. These values range from 0.05 to 1.1 m. (**D**) Levee breaches across historical marsh channels to facilitate tidal connectivity in salt ponds are shown in blue. (**E**) Current and (**F**) restored marsh habitat types, determined by elevation relative to tidal datum, as shown in Eq. 1. The three shades of green, from dark to light, denote low, transition, and high marsh habitat. Created with ArcMap 10.7.1 (https://desktop.arcgis.com/en/quick-start-guides/10.7/arcgis-desktop-quick-start-guide.htm).

Within California, San Mateo County has the most projected flood exposure due to climate change, with more than 140,000 people and $50 billion of property exposed to flooding through this century^6^. Communities are already beginning to experience these impacts. San Mateo County’s bay shoreline (Fig. 1B) is highly altered and contains critical public infrastructure, including the San Francisco International Airport (SFO, Fig. 1B), touchdowns of two regional bridges, an interstate highway, the heart of the Silicon Valley technology industry, and over 750,000 residents. Levees are an essential component of flood control throughout the region^8^. Much of the urban coast is built on historical wetlands that have been drained, diked, and filled; wetlands have also been diked to create ponds for industrial salt production^9^. The result of this filling, diking, and development of former tidal marshes is that San Mateo County is one of only six counties in the country with over 100,000 residents at risk of flooding with 0.9 m of SLR^10^.

Communities in San Francisco Bay rely heavily on gray infrastructure (primarily levees and seawater pumping) for flood protection today, but a wide variety of pathways for integrating nature-based shorelines have been identified regionally^11–13^. There is strong interest in the use of wetlands in reducing flooding—in 2016, Bay Area voters passed Measure AA, which uses a $12 per year parcel tax to restore wetlands to prepare for climate change^14^ in addition to providing other co-benefits including supporting fish and wildlife and carbon sequestration^15–18^. However, coastal development has altered or removed up to 90% of the Bay’s historical tidal wetlands^19^, resulting in dramatic habitat loss and fragmentation. In San Mateo County, large marshes in the northern part of the county were buried and developed to create planned communities; in the southern part of the county, marsh habitat was diked and drained to create ponds for salt production. In the past several decades, marsh restoration has emerged as a unifying management objective in the Bay, particularly restoration of legacy salt ponds^20–22^. San Mateo County’s tidal marshes are among the highest ranked for conservation priority across the Bay^23^. Marshes receive sediment supply from suspended sediment deposition during periods of flooding, a process that facilitates vertical accretion^24^. A large portion of San Mateo County’s bay coast is historical marsh that has been diked to prevent tidal flooding^25^; many historical diked marshes have subsided in elevation and are no longer high enough to support a full range of marsh vegetation. Marsh restoration in the region usually includes strategic breaching of levees across historical channels (Fig. 1D) to allow inundation and sedimentation to occur in diked historical marsh (Fig. 1C), facilitating revegetation by native marsh plants (Fig. 1E,F).

With the highest future flood risk in the state and large areas of wetlands with potential for restoration, San Mateo County’s shoreline is a key study site for investigating the potential of marsh restoration as a nature-based flood defense. Few studies have quantified the flood risk reduction benefits provided by habitat^26^. Even fewer assess benefits of habitat restoration^27–30^ and none have rigorously quantified the value of restored habitat under climate change. Impacts of climate change present pressing challenges to coastal communities and assessments of the benefits of both natural and artificial infrastructure will need to account for these challenges. Here, we address this gap by assessing the flood risk reduction benefits of marsh restoration projects in San Francisco Bay, under current and future climate conditions, to characterize how and where they provide climate adaptation benefits now and into the future.

## Results

Overall, marsh restoration decreases flood risk, providing a net positive annual expected benefit (AEB) and present value in avoided damage. These benefits increase with SLR; benefits increase by a factor of 5 with 0.5 m SLR and by a factor of more than 20 with 1.0 m SLR (Table 1). Population protected by restoration is also shown in Table 1. At present sea level, a large portion (43%) of the people who would be protected from flooding due to restoration live in socially vulnerable census block groups. As sea levels rise, the percent of population that is protected by restoration and that is socially vulnerable decreases, a trend driven by SLR causing the floodplain to expand to higher elevations and less socially vulnerable census block groups.

**Table 1:** (**A**) Property and (**B**) people protected by marsh restoration in San Mateo County under 3 different sea level rise (SLR) scenarios. Present value is calculated assuming a 50-year project lifespan, with discount rates of 4, 7, and 10%.

SLR [m]	A				B	
	Annual expected benefit [$ millions]	Present value [$ millions]			Number of people who benefit from flood reduction	% of benefits in census block group ranked high or highest social vulnerability
		Discount rate				
		4%	7%	10%		
0	1.5	33.2	21.3	15.3	7	43%
0.5	7.6	164.4	105.6	75.9	322	18%
1	36.2	777.2	499.3	358.7	254	9%

**Figure 2:**
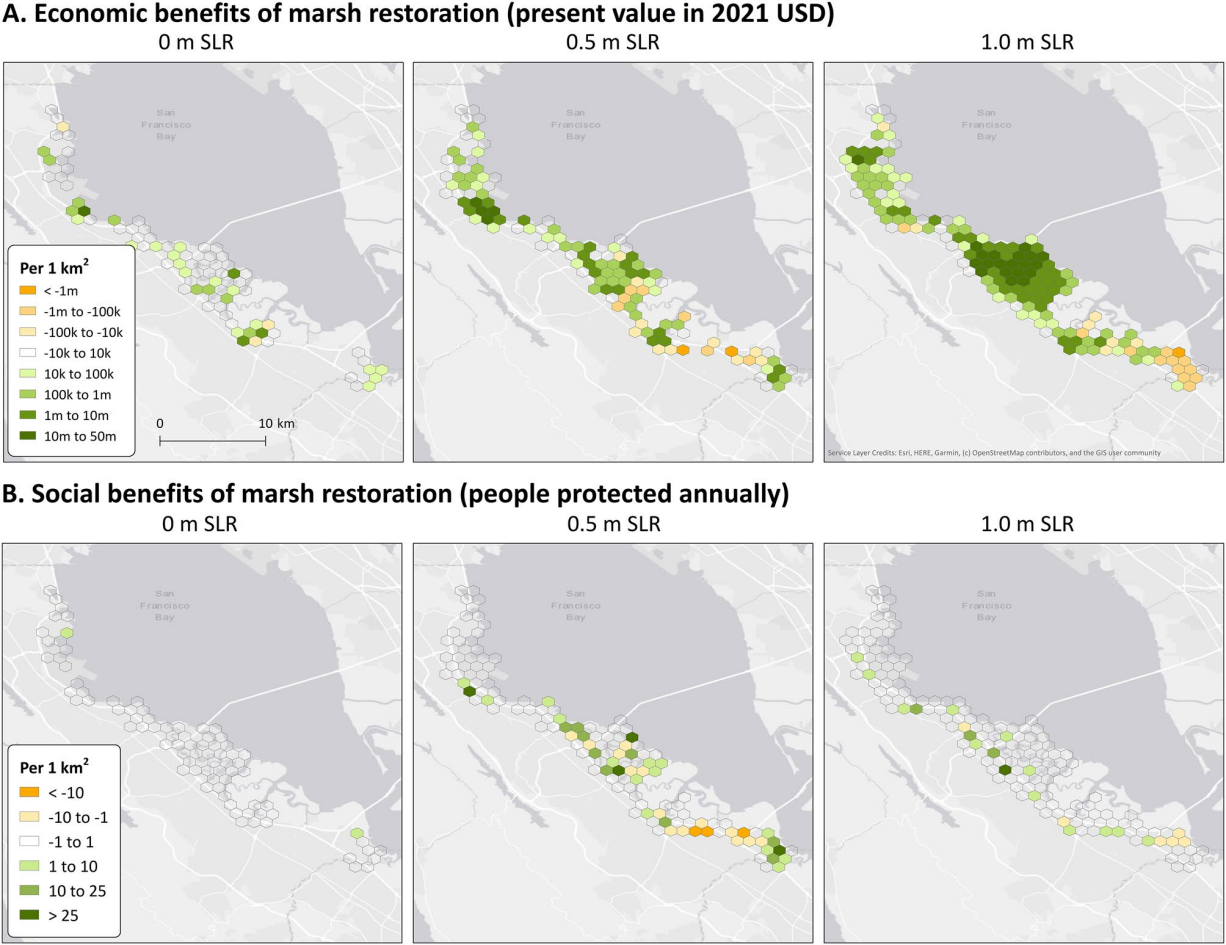
Spatial distribution of (**A**) economic and (**B**) social flood reduction benefits of marsh restoration with sea level rise. Green colors signify positive present value and people protected while orange colors signify negative present value and increased risk. Created with ArcMap 10.7.1 (https://desktop.arcgis.com/en/quick-start-guides/10.7/arcgis-desktop-quick-start-guide.htm).

Although the present value of marsh restoration is positive, the benefits vary spatially (Fig. 2). Flood depth reduction benefits are not always directly attributable to a certain restoration site due to complex hydrodynamics, such as channels, creeks, and sloughs. However, the restoration south of SFO, a simple fringing marsh restoration, provides directly attributable benefits. The restoration, which is 14.7 ha in area, produces a present value of $12.7 million in flood reduction benefits (at a 7% discount rate), i.e., $900,000/ha, a value that can be used by planners and managers as a “break-even point” for the cost of restoration. The value of restoration at this site increases substantially with 0.5 m of SLR to $9.2 million/ha.

**Figure 3:**
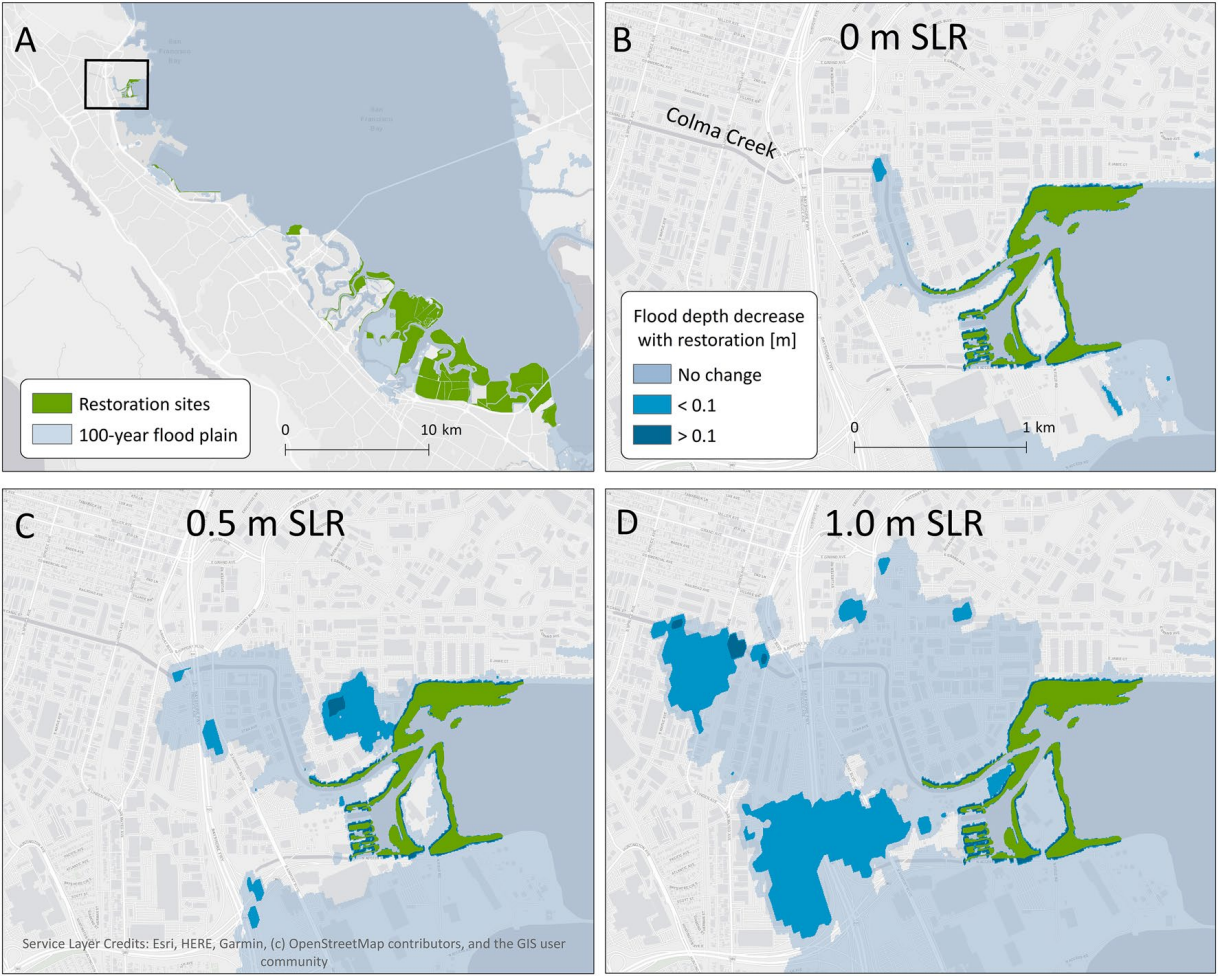
Effects of marsh restoration and climate change in Colma Creek. (**A**) shows the study region. (**B**–**D**) show reduction in flood heights during a 100-year storm due to habitat restoration with 0, 0.5, and 1.0 m SLR. The green areas represent locations of marsh restoration, the pale blue areas show flood extent, and the bright blue areas represent regions where flood depth is decreased due to the marsh restoration. Created with ArcMap 10.7.1 (https://desktop.arcgis.com/en/quick-start-guides/10.7/arcgis-desktop-quick-start-guide.htm).

Levee height relative to storm water levels is a key determinant of whether a restoration will provide risk reduction benefits. In many locations in the study region and in San Francisco Bay more broadly, marshes front levees. Areas protected by levees will only flood when levees overtop—thus, restoring a marsh on the seaward side of a levee can only reduce flood risk if the levee is at risk of overtopping. This outcome is illustrated by the 100-year flood plain with different sea level scenarios at the mouth of Colma Creek, in the northern end of the study region (Fig. 3). With 0 m SLR, the restoration provides limited flood depth reduction benefits because the levees lining the creek do not overtop and so there is limited flooding (Fig. 3B). However, as SLR progresses, water levels from storms will exceed levee height, causing flooding in the lee of the restoration. Therefore, the restoration provides increasing benefits over time (Fig. 3C,D). The marsh at Colma Creek, if restored at present sea level, will provide increasing benefits as levees are increasingly overtopped, even as vegetation in the restored marsh restoration migrates upslope where there is space to do so.

## Discussion

We quantified the flood risk reduction benefits provided by marsh restoration under several climate change scenarios in San Mateo County. Overall, marsh restoration provides meaningful flood reduction benefits. The highest value restoration site in our study is a narrow fringing marsh restoration south of San Francisco International Airport (SFO), which provides a present value of $900,000/ha of restored marsh with 0 m SLR and $9.2 million/ha with 0.5 m SLR (Fig. 2). Countywide, flood reduction benefits increase by a factor of 5 and 23 with 0.5 m and 1.0 m of SLR, respectively. These values are encouraging for managers who may be concerned about the lifespan of marsh restorations in an era of accelerating SLR. Our results indicate that annual benefits will increase into the future as SLR progresses, highlighting high potential benefits for investments in adaptation now.

Results from this analysis can help to prioritize where investments should be made in marsh restoration for flood reduction. In this study system, simple and smaller restorations provide more flood reduction benefit, as measured by amount of avoided damage. Restoration of fringing marshes could be considered as an alternative or a supplement to gray infrastructure solutions as communities adapt to impacts of climate change. Results indicate that restoration of diked and drained historical marshes, such as the salt ponds in this study region, may not provide straightforward flood reduction benefits. Determining how such sites can be restored to produce ecological benefit and flood risk reduction benefit warrants further study. If managers and communities are interested in habitat restoration in the salt ponds, raising levee heights behind the ponds could be considered as part of a green-gray infrastructure solution to restore habitat while keeping adjacent communities safe. These results, along with other work^22^, highlight the enduring cost of diking and draining marsh habitat, particularly in an era of accelerating SLR.

We simulated a total of 2,937 ha of marsh restoration. Excluding restoration of large offshore islands and current and former salt ponds, which provide the least clear benefits^31^, the total area of restoration is 367 ha. Based on a conservative estimate of restoration costs in developed countries, restoring this area of marsh would cost on the order of $70 million, or about $200,000 per hectare (in 2021 USD)^32^, which is an order of magnitude lower than present value of restoration with 1.0 m SLR. In contrast, previous work indicates that the cost of raising coastal protection structures along the bay coast of San Mateo County (calculated by determining the % of Bay shoreline in San Mateo County, which is ~ 12%) to prepare for the highest SLR scenario we explored, 1.0 m, would be $7–18 billion (in 2021 USD)^7^. Certain marsh restorations provide higher present value than others, reaching up to $900,000/hectare with present day sea level in one instance. Values like this can be weighed against the cost of implementing the restoration to assess whether investing in marsh restoration can yield positive returns in avoided damages.

The restoration scenarios we explored here do not fully protect the county from flooding, but they do meaningfully decrease flood risk. These scenarios could also decrease the height and, thus, cost of shoring up existing hard infrastructure^33,34^, and cost less than 1% of preparing gray infrastructure for 1.0 m of SLR. Although marsh restoration is unlikely to be a silver bullet solution for communities interested in increasing resilience to SLR and storm-driven flooding, this work demonstrates it can be a part of the solution while simultaneously providing community and ecosystem co-benefits.

Future research should focus on wave-resolved interactions between vegetation, levees, and waves. In this study region, a densely urbanized estuarine coastline with the some of the highest future flood risk in the nation, marshes tend to be on the seaward side of levees. In such settings, we demonstrate that marsh restoration can influence flood depths when levees overtop, however, when levees do not overtop the influence of restoration is limited to reducing damage to the levee system itself. While our work includes phase-averaged (spectral) wave dynamics, SWAN does not model individual waves or wave-wave interactions that are necessary to quantify swash, a key driver of levee overtopping, as well as levee damage. Previous work indicates that marshes can meaningfully reduce dynamic water levels at levees^33^, which, paired with these results, demonstrate that a wave-resolved investigation of the interactions between levees and marsh vegetation may give insight to how marsh restoration could influence levee overtopping and hydraulic failure.

Our value of benefits is conservative because it does not include additional co-benefits. A valuation of ecosystem services of marsh restoration in northern San Francisco Bay reports that fluvial and storm surge driven flood reduction accounts for 86% of ecosystem service benefits; recreation and water quality improvements account for the remaining 14% of benefits^35^. If these percentages hold true in San Mateo County, the total benefits of restoration with 0 m SLR would increase from $21 million to $25 million.

Our modeling is conservative because we have not accounted for future changes in marsh height or shape. As sea levels rise, marshes may either vertically accrete, retreat landward, erode, prograde, or drown, depending on sediment supply, location, and morphology. In this study, we assumed that marsh vegetation will retreat upslope and that sites will be squeezed with SLR and transition to mudflat but will otherwise remain the same into the future. This assumption neglects the possibilities of sediment and organic matter accretion on the marsh platform and allowing the marsh elevation to keep up with SLR and maintain vegetation coverage, which could be incorporated using a Marsh Equilibrium Model (MEM)^36^, though confirmation of this possibility would require detailed marsh biomass measurements and precise marsh elevation measurements, presenting challenges in a regional two-dimensional domain. While this type of regional analysis was not possible here, radioisotope dated cores estimate marsh accretion rates of 0.2–0.5 cm/year in San Francisco Bay, suggesting that marshes have been keeping up with sea level rise over the last 150 years^15^. However, current accretion rates are less than 4% of what will be necessary for marsh elevations to keep pace with accelerating sea level rise by 2100^37^. For example, Laumeister Marsh, a presently healthy study site in San Mateo County, is predicted to completely lose high marsh habitat by 2100^38^. Findings like these highlight the need for sediment nourishment programs like those described in this work.

The assumption of no future change in marsh morphology also neglects the possibility that the marsh platform will laterally erode or prograde as water levels rise. Surveys indicate that over recent decades, marshes in the northern part of San Francisco Bay, which is more wave exposed than our study region, have exhibited both spatial and temporal variability in edge change on a local scale^39^. Management interventions to stabilize a marsh edge, such as restoration of oyster reefs, have been shown to be effective in decreasing marsh-edge erosion^40^. Some form of marsh-edge stabilization will likely be necessary in the simulations presented here, particularly those including future SLR.

Our results show that marsh restoration can be a cost effective approach to climate adaptation in one of the most at-risk counties in the U.S. Considering future climate scenarios is becoming common practice with traditional infrastructure solutions^41–43^, and similar approaches should be taken when assessing the potential of nature-based solutions. This work demonstrates that investments in nature and community resilience made today can result in increasing payoffs as climate change progresses and risk increases.

## Methods

### Model description

The Delft3D Flexible Mesh modeling suite^44^ and Simulating WAves Nearshore^45^ were two-way coupled to assess the flood impacts of marsh restoration. Delft3D FM solves the shallow-water equations on staggered unstructured grids in one-, two-, and three-dimensional schematizations with a finite volume method. Here, we ran the model in a two-dimensional-horizontal schematization, which simulates depth-averaged flow. SWAN, a third-generation, phase-averaged numerical wave model, simulates the propagation of waves from deep to shallow waters by solving the energy balance and action balance equations. The use of the energy spectrum through these equations allows models such as SWAN to significantly reduce computational time, but as a result, they do not resolve individual waves. Wave-current interactions are simulated in the coupled model, and result in changes in current refraction, bottom friction, wave setup, turbulence, and bed shear stress. Thus, this model includes tides, steric changes in water level, creek discharge, wave setup, and relative SLR. This model does not account for swash, which requires a wave-resolving model.

The domain for this model was adapted from previous regional modeling^46^, and the approach was based on the Coastal Storm Modeling System^4,47^. The open boundary was perpendicular to the main bay channel, approximately 5 km south of the Bay Bridge. The grid resolution was increased over existing and potential marsh habitat to ~ 10 m, to capture complex bathymetry and tidal channels of the marshes (Figure S1). Bathymetry was derived from a LiDAR Elevation Adjustment with NDVI (LEAN)-corrected 5-m horizontal resolution digital elevation model, used above -1 m NAVD88^48^. The LEAN-correction facilitates the LiDAR collected elevation data to capture the mudflat rather than the top of marsh plants. 10-m resolution model was used below -1 m NAVD88^49^. The tidal constituents at the open model boundary for this model are derived from the FES Global Tide Model^50^. Constituents were calibrated to maximize model-data agreement.

Land cover type was determined the National Land Cover Database^51^, which details satellite-derived land cover nationally. Open water Manning’s friction values were set to 0.02^46^ and supported by sensitivity testing. Vegetation was represented implicitly with Manning’s friction coefficients of 0.03, 0.035, and 0.04 for low, transition, and high marsh zones, respectively^52,53^.

Tidal vegetation location in the Bay Area Aquatic Resources Inventory^54^ was used to determine marsh habitat locations. Because marsh plants are sensitive to flooding periods^55^, the extents of the three different marsh vegetation zones were determined based on elevation relative to tidal data (mean sea level, or MSL, and mean higher high water, or MHHW). This was done using z*^11^ as a metric: $z^{*} = \frac{elevation-MSL}{MHHW-MSL}$. Areas with a z* value between 1.02 and 1.38 were assumed to support high marsh; locations with a z* value between 0.75 and 1.02 were assumed to support transition marsh; locations with a z* value between -0.14 and 0.75 were assumed to support low marsh^11^. MHHW and MSL were determined from values reported in Appendix 5 of Beagle et al.^11^ and assumed to be constant across the domain. This assumption neglects the regional < 20 cm variation in MHHW. MSL was assumed to be 1.00 m NAVD88 and MHHW was assumed to be 2.12 m NAVD88. Flood control infrastructure was included as a sub-grid feature with location determined from the San Francisco Estuary Institute’s Bay Shore Inventory^8^, and height determined from the most highly resolved DEM available^56^. The two gauged creeks in the study region are San Francisquito Creek and San Mateo Creek (Figure S1), and both were included in the model, allowing for the simulation of flooding driven by high offshore water-levels as well as upland flooding driven by high creek flows.

### Calibration and validation

To calibrate the model, non-tidal residuals (NTR) at the model boundary were derived from a previously develoed 70-year model hindcast^46,57^. This hindcast, which spans from 1950 to 2020, was forced with tides and ERA5 30-km resolution winds^58^, which provide a longer temporal record than other hindcasts of finer resolution, facilitating a more robust calibration. A low pass filter was used to remove the tidal signal from the 70-year time series, and to generate the NTR signal, which was used to force Delft3D. For calibration and validation, waves were simulated with the built-in fetch-limited wave formulation, which is more computationally efficient than SWAN, and forced with Rapid Refresh wind hindcasts^59^. This data is resolved to 13 km, which is lower than many other hindcast winds, but it is the only available data for the study region during the calibration period. Flow rates for San Mateo and San Francisquito creeks (Figure S1) were derived from U.S. Geological Survey gauges for the calibration period of 2009 to 2011^60^.

Modeled water-levels were compared to measurements collected by the NOAA Redwood City Tide Gauge^61^ and by a water logger deployed in Laumeister Marsh during storms in 2010 and 2011^62^. The months of January 2010 and March 2011, which included significant storms and extreme high water-levels, were used for calibration and validation, respectively. The amplitude and phase offset of individual tidal constituents were isolated from the total water level time series using a harmonic analysis^63^ and compared between the modeled and observed data. The differences were used to adjust to the FES constituents on the boundary to improve model data agreement (Figure S2 and S3). Root mean square error (RMSE) for the calibration period was 9 and 12 cm at Redwood City and Laumeister Marsh, respectively, and 10 and 20 cm at Redwood City and Laumeister Marsh, respectively, for the validation period. The full tidal amplitude at both sites is about 2.5 m and 1.5 m, respectively.

Model-observation agreement was initially very poor in Laumeister Marsh because the marsh channel in which observations were taken was unresolved in the 5-m resolution DEM and the 10-m resolution model grid. Thus, to facilitate model-observation comparison in Laumeister Marsh, channel depth was increased manually. Simulations with varying marsh platform height and marsh channel depth were done during the calibration period to assess the model sensitivity to marsh bathymetry. This analysis revealed that model data agreement in Laumeister Marsh is most strongly sensitive to channel depth, and less sensitive to platform elevation. Results from the sensitivity analysis are shown in Table S1. After manually adjusting channel depth, a systematic bias was still present in the Laumeister Marsh model-data agreement. This bias, which was not corrected, is likely due to uncertainty in marsh channel depth in Laumeister Marsh.

### Hydrodynamic simulations

To understand how marsh habitat restoration influences flood depth and extent, simulations with existing marshes and potential restoration projects were compared under various sea level and storm conditions, including: 0, 0.5, and 1.0 m of SLR, within the range of most updated intermediate estimates for California by 2100^1^, along with annual, 20-year and 100-year storm conditions. For each of these SLR scenarios, several marsh habitat distributions were simulated, including existing marsh^54^, and an extensive regional restoration. The simulated marsh restorations were identified in close collaboration with local flood managers over a series of workshops, with the goal of developing plausible scenarios of marsh restoration to assess effects on flood risk in the study region.

Storm water levels and wind and pressure time series were derived from Barnard et al.^47^ and Barnard et al.^4^. These were originally developed for the Coastal Storm Modeling System (CoSMoS). A twenty-first century total water level proxy time-series was created from global climate model sea surface temperatures, winds, sea level pressures, and water levels from this time series were ranked. Mean, annual, 20-year, and 100-year events occurring on future dates were extracted from the ranked water levels. Local models were forced with downscaled twenty-first century wind and pressure for the future dates. These storms were run in combination with plausible twenty-first century increases in sea level, thus capturing non-linear interactions between waves and water levels. These downscaled twenty-first century storms were used as boundary conditions in the present work.

Data from the tide gauge closest to the open boundary, located in Alameda^61^, were appended before and after the 50-h storm water level time series at the open boundary, to facilitate isolating NTR from changes in water level due to tides. A low-pass filter was used to derive the NTR. This caused the storm water levels to become non-progressive in the second day of the simulation, so water levels from the first day of the simulation were used to determine maximum flood depth.

Creek flows for San Francisquito Creek were determined from flow rates for eight California creeks and rivers coincident with annual, 20-year, and 100-year twenty-first century storms^64^ based on a twenty-first century time series of flow for the Sacramento/San Joaquin Delta^65^. San Mateo Creek was not included in that analysis, so flow rates for San Mateo Creek were determined by the same percentile of flow from the stream gauge as flow rates from San Francisquito Creek, which is 20 km away. This approach neglects localized watershed differences in normalized flow rates.

The study region is home to low-lying marshes, which, though presently classified as marsh habitat, are too low in elevation to support a full range of marsh vegetation. In such locations, the restoration projects assume sediment nourishment and re-vegetation that would bring the sites to a mean elevation of 2 m NAVD88, or + 1 m relative to regional MSL, which is representative of healthy marshes in San Francisco Bay^55^. The uniform elevation increase was applied to sites that were identified by stakeholders as potential restoration sites (Fig. 1C). Vegetation zones were shifted according to the new z* score (Fig. 1E and Fig. 1F). For the 0.5 and 1.0 m SLR scenarios, vegetation zones were modified to represent the corresponding upward and landward shift of marsh vegetation zones caused by an increase in mean sea level. The simulated restorations also included breaching several levees surrounding salt ponds. We used satellite and historical photography, as well as conversations with stakeholders, to determine locations for the breaches, which are made across historical channels (Fig. 1D).

The restoration sites and projects were implemented using the most recently available data on marsh distribution and levee location. However, certain sections of the region’s levee system have been breached since the San Francisco Estuary Institute Bayshore Inventory was published in 2016 and a section of Bair Island (Figure S1) has been restored to marsh habitat from open water in recent years. To ensure hydrodynamics in the model reflect the current state of the study region, we manually edited the datasets for marsh distribution and levee elevation based on stakeholder input.

### Stakeholder workshops

Stakeholder engagement was facilitated by The Nature Conservancy (TNC). TNC has used market-based tools to advance conservation in California with extensive partnerships with municipal lanners, local and state agencies, and risk industry partners across the state and in the Bay Area. The stakeholder group met six times from beginning (scenario design) to end (final results) of the project and included 10 + representatives from: The San Francisco Estuary Institute, San Mateo County, the San Francisquito Creek Joint Powers Authority, the San Mateo County Flood & Sea Level Rise Resiliency District (One Shoreline), the California State Coastal Conservancy, the San Francisco Bay Metropolitan Transportation Commission, the California Department of Insurance and SwissRE. With the stakeholder group we gathered information on many topics including conservation goals in San Francisco Bay, local best practices for marsh restoration, challenges faced during previous restoration projects, and flood history within the study area. We developed restoration scenarios and made iterative changes based on group feedback and modified our analysis approach based on implementation opportunities identified by stakeholders.

### Flood damages and socioeconomic analysis

To calculate flood damages on buildings, we used the Federal Emergency Management Association’s (FEMA’s) Flood Assessment Structure Tool (FAST)^66^. FAST connects FEMA’s Hazus depth-damage curves with flood maps and built infrastructure inventories to quantify flood damages. Depth-damage curves quantify damage to a structure as flood depths increase, using curves that vary by structure type. The exposure data was obtained from the National Structure Inventory (NSI)^67^ which includes information on structure replacement cost, content cost, material type, basement type, and number of floors. NSI includes about 200,000 structures in San Mateo County. NSI and the flood depth maps produced from the hydrodynamic model were used as inputs to FAST, which determines the appropriate depth-damage curve for each structure and the resulting economic damage. Damages across all structures in the domain were aggregated to quantify economic impacts of flooding. Flood damages were calculated with and without the restoration projects.

The damages associated with each return period flood zone were used to determine the annual expected damage, which represents the frequency-weighted sum of damages for the full range of flood events and is a measure of flood risk per year^68^: ${Annual}\; {expected} \;{damage}\; \left(AED\right)= \frac{1}{2}{\sum }_{i=1}^{n}\left(\frac{1}{{T}_{i}}-\frac{1}{{T}_{i+1}}\right)\left({D}_{i}+ {D}_{i+1}\right)$, where *i* represents the number of return periods, *T*_*i*_ represents the return period, and *D*_*i*_ represents the damage associated with a storm with a return period of *T*_*i*_, following the formula for trapezoidal integration. The difference in damage between the baseline flooding and the flooding with habitat scenarios determines the avoided damages provided by the restoration projects. The annual expected benefit (AEB) of the restoration projects can be calculated as the differences with the baseline situation (without restoration):

$${Annual} \;{expected}\; {benefit}\; \left(AEB\right)= {AED}_{existing}-{AED}_{restored}.$$

 To discount future benefits of adaptation investments^26^, the present value of the projects was determined by summing annual expected benefit over the lifetime of the project, represented by *n*, and discounting the benefits of each future year by *i:* ${Present} \;{Value}\; \left(PV\right)= \sum \frac{{AEB}_{n}}{{\left(1+i\right)}^{n}}$. We assumed the project lifespan to be 50 years and included several discount rates^26^. 7% is the standard value used by the U.S. Army Corps of Engineers.

The number of people affected by impacts of marsh restoration on flooding in San Mateo County was determined following a similar approach. Population across each census block group^12^ was assumed to be evenly distributed between residential buildings, and population impacted was determined by whether a residential structure was flooded. Thus, for determining the social impacts of marsh restoration, the formula for annual expected damage was used, where *D*_*i*_ = residences flooded per census block group × population of CBG ÷ total residences in CBG. Absolute and percent in social and economic risk with habitat restoration and without can be found in Table S3.

The San Francisco Bay Conservation and Development Commission’s Adapting to Rising Tides project has ranked social vulnerability of census block groups based on demographics^12^. Social vulnerability of census block group was determined through 12 indicators (Table S2). In this work, we considered most vulnerable census block groups to be those marked as “high social vulnerability” and “highest social vulnerability”.

### Supplementary Information

Supplementary Information.

## Data Availability

All data and code are available upon request from corresponding author.
