# Supplementary material for: The value of marsh restoration for flood risk reduction in an urban estuary
Source: Sci Rep. 2024 Mar 21;14:6856. doi: 10.1038/s41598-024-57474-4 (PMC10957971; doi:10.1038/s41598-024-57474-4)
Supplement: Supplementary file 1 — Supplementary Information. [file 41598_2024_57474_MOESM1_ESM.docx]

# Supplementary Information

## Figures and tables

**Figure S1.** Depiction of the model mesh shown in red. The yellow and blue circles show locations of calibration and validation data. The purple and green circles show locations of creek gauges that were used to determine fluvial flow in San Franciscquito and San Mateo Creeks, respectively. The text boxes in the mesh denote the grid resolution of the mesh in its lowest and highest resolution, which are the middle of the bay and in existing and potential San Mateo County marshes, respectively. Created with ArcMap 10.7.1 (https://desktop.arcgis.com/en/quick-start-guides/10.7/arcgis-desktop-quick-start-guide.htm).


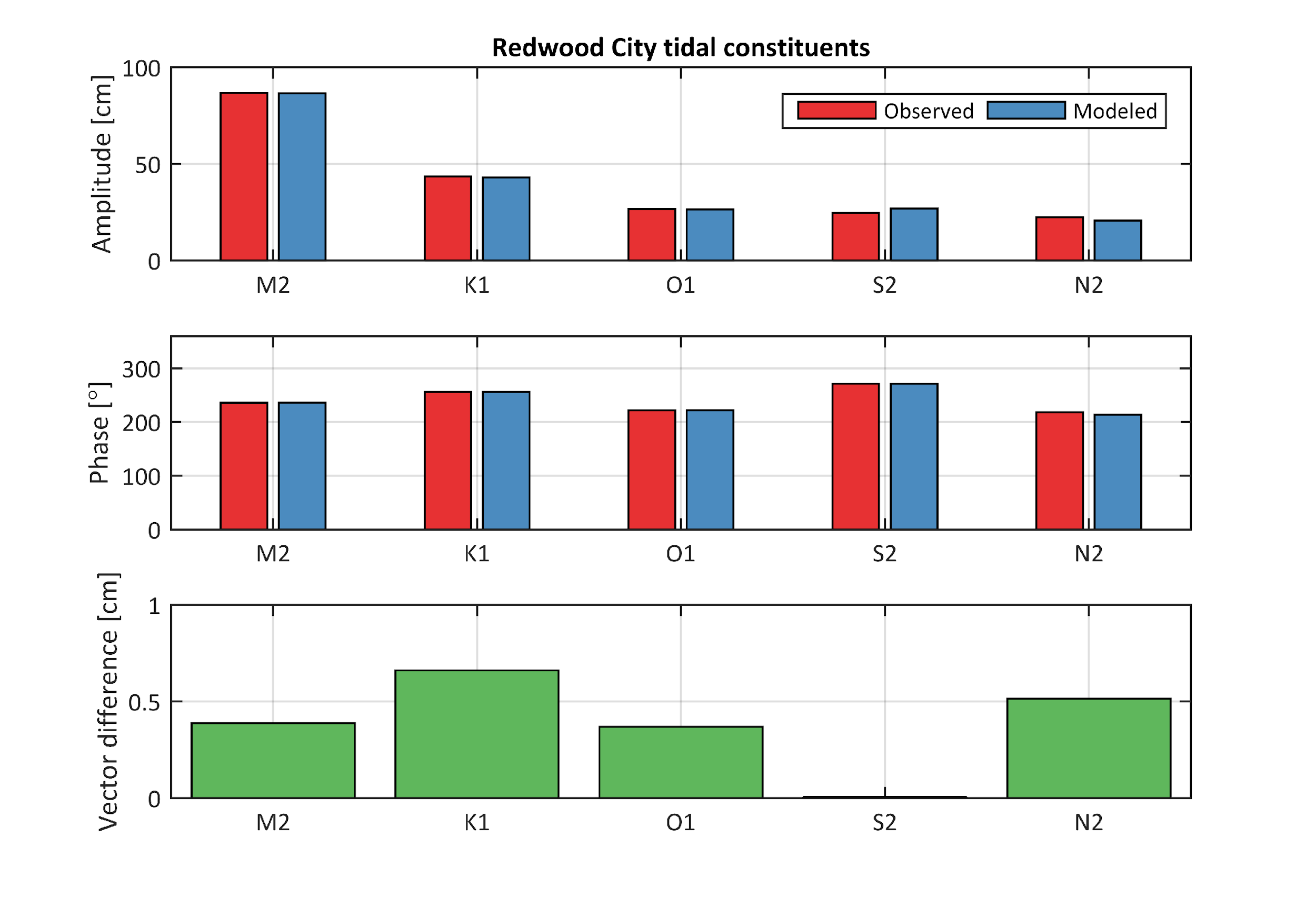


**Figure S2.** Observed (red) and modeled (blue) amplitude (cm, row 1) and phase (degrees, row 2) of the top five tidal constituents in the model; vector difference between observed and modeled tidal constituents (cm, row 3).


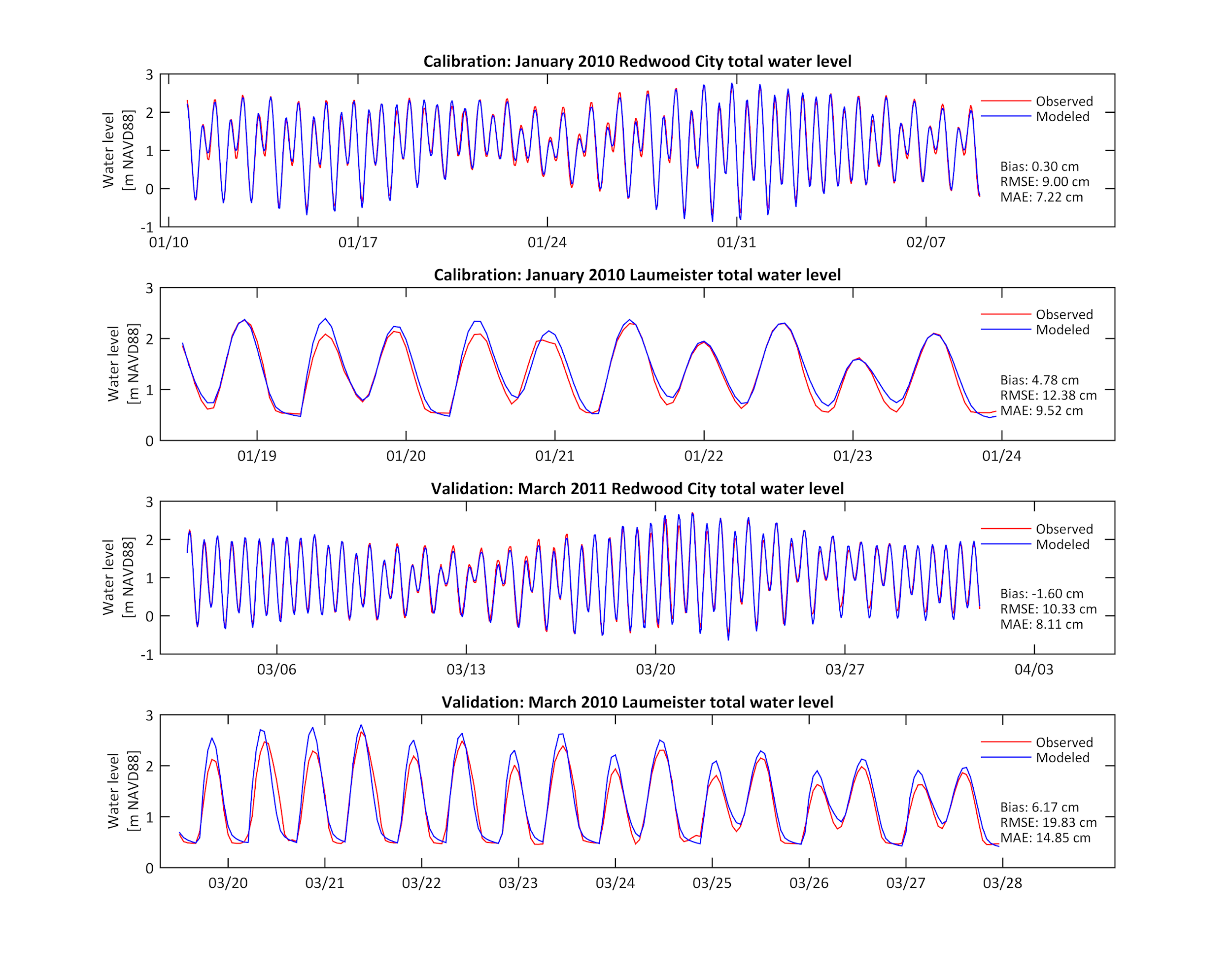


**Figure S3.** Calibration (rows 1 and 2) and validation (rows 3 and 4) time series, comparing model data (blue) and observations (red) at the Redwood City tide gauge (rows 1 and 3) and at a water logger deployed in Laumeister Marsh (rows 2 and 4). Skill scores include bias, root mean square error (RMSE) and mean absolute error (MAE).

**Table S1.** Results from sensitivity analysis of water levels on bathymetry in Laumeister Marsh. Results show that modeled water levels in the marsh channel are highly dependent on local channel depth and slightly dependent on local marsh platform height. Remote bathymetry has limited impact on model performance. Root mean squared error (RMSE), bias, and mean absolute error (MAE) of water levels were used to evaluate model performance.

| **Bathymetry** | | **Laumeister water levels** | | | | **Redwood City water levels** | | |
| --- | --- | --- | --- | --- | --- | --- | --- | --- |
| **Channel Depth** | **Platform Height** | **RMSE (cm)** | | **Bias (cm)** | **MAE (cm)** | **RMSE (cm)** | **Bias (cm)** | **MAE (cm)** |
| Deep^*^ | High^+^ | 16.63 | 6.38 | | 13.55 | 9.24 | 0.35 | 7.35 |
| Deep | Mid^++^ | 15.65 | 6.23 | | 12.65 | 9.24 | 0.35 | 7.35 |
| Deep | Low^+++^ | 15.16 | 6.17 | | 12.05 | 9.23 | 0.36 | 7.34 |
| Mid^**^ | High | 43.76 | 20.50 | | 34.82 | 9.24 | 0.35 | 7.35 |
| Mid | Mid | 41.87 | 20.02 | | 33.18 | 9.24 | 0.35 | 7.35 |
| Mid | Low | 41.00 | 19.99 | | 32.19 | 9.23 | 0.36 | 7.34 |
| Shallow^***^ | High | 107.03 | 63.03 | | 91.00 | 9.42 | 0.34 | 7.48 |
| Shallow | Mid | 101.79 | 59.55 | | 85.59 | 9.42 | 0.34 | 7.48 |
| Shallow | Low | n/a | n/a | | n/a | n/a | n/a | n/a |

^*^ 0.25 m NAVD88; ^**^1 m NAVD88; ^***^2 m NAVD88; ^+^2.73 m NAVD88; ^++^2.23 m NAVD88; ^+++^1.73 m NAVD88

**Table S2**. Indicators and rank of social vulnerability as determined by BCDC Adapting to Rising Tides “Vulnerable Communities”^1^.

| **Social Vulnerability Indicators** | **Social vulnerability rank determination:**  Number of indicators in a given percentile relative to the 9 county Bay area |  |
| --- | --- | --- |
|  |  |  |
| Very low income | **Highest social vulnerability:**  8 or more indicators in the 70th percentile  OR  6 or more indicators in the 90th percentile |  |
| Not a U.S. citizen |  |  |
| Without a vehicle |  |  |
| People with disability |  |  |
| Single parent households | **High social vulnerability:**  6-7 indicators in the 70th percentile  OR  4-5 indicators in the 90th percentile |  |
| Communities of color |  |  |
| Limited English proficiency |  |  |
| Without a high school degree |  |  |
| Young children under 5 | **Moderate social vulnerability:**  4-5 indicators in the 70th percentile  OR  3 indicators in the 90th percentile |  |
| Severely housing cost burdened |  |  |
| Older adults |  |  |
| Renters |  |  |

**Table S3**. On top, annual expected damages (property loss and people flooded) with existing and restored habitat under 3 different SLR scenarios. On the bottom, total and percentage of protected people and property with existing and restored habitat under 3 different SLR scenarios. Property protected increases continuously with sea level rise because it is calculated across the floodplain with depth damage curves, such that all structures within the floodplain can contribute to the total value of property protected, providing that restoration decreases the flood depth a structure experiences. Population protected does not increase continuously with sea level rise because it is calculated by determining residences that are flooded with the existing landscape but are not flooded with marsh restoration. Thus, the population protected does not cumulatively increase, but rather shifts towards the edge of the floodplain as sea levels rise.

|  | Existing habitat | | Restored habitat | |
| --- | --- | --- | --- | --- |
| SLR | Property loss  ($ millions) | Population flooded | Property loss  ($ millions) | Population flooded |
| 0 | 366 | 13588 | 364.80 | 13581 |
| 0.5 | 1,020 | 29576 | 1,012.22 | 29350 |
| 1.0 | 5,839 | 112668 | 5,802.92 | 112450 |
|  |  |  |  |  |
| SLR | Total property protected  ($ millions) | Total population protected | % Property protected | % Population protected |
| 0 | 1.55 | 7 | 0.4 | 0.1 |
| 0.5 | 7.68 | 226 | 0.8 | 0.8 |
| 1.0 | 36.24 | 218 | 0.6 | 0.2 |

## Discussion of uncertainties in the flood risk model

Uncertainty is introduced into this model at numerous points. The main uncertainty sources of analyses like this involve the hydrodynamic modelling, bathymetry, elevation, damage models and socioeconomic changes^2^. This model includes local defense structures, but results are sensitive to the height of the levees in the model. Thus, the hydrodynamic analysis can be considered conservative in the difference between existing and marsh restoration scenarios, but the absolute flood results may be non-conservative in certain areas, such as behind levees where the crest height is not fully resolved. There may be unresolved hydraulic connectivity in some places, which could result in an underestimate of flood extents. Studies specifically focused on characterizing the uncertainty in coastal flood damage models at local scales have determined that the greatest sources of uncertainty are the elevation model and the depth-damage functions^3,4^. This analysis relies on bathymetric and topographic data at the highest resolution available for San Francisco Bay. The damage curves for each building type correspond to the official curves included with FEMA-HAZUS, which were developed with local empirical data on building damage vulnerability for the United States. Other additional uncertainty factors affecting the flood results include the forcing conditions (waves, sea levels and storms duration) and differences in the spatial distribution of building stock and exposure values.

# References

1. BCDC, Caltrans, MTC, ABAG & BARC. Adapting to Rising Tides Bay Area, Regional Sea Level Rise Vulnerability and Adaptation Study. 205 (2020).

2. Reguero, B. G. *et al.* The value of US coral reefs for flood risk reduction. *Nat. Sustain.* **4**, 688–698 (2021).

3. Menendez, P., Losada, I. J., Torres-Ortega, S. & Beck, M. W. Assessing the effects of using high-quality data and high-resolution models in valuing flood protection services of mangroves. *PLoS One* 1–14 (2019) doi:10.17605/OSF.IO/S5V7K.

4. Parodi, M. U. *et al.* Uncertainties in coastal flood risk assessments in small island developing states. *Nat. Hazards Earth Syst. Sci.* **20**, 2397–2414 (2020).
